# Assessing seasonal spatial segregation by age class of beluga whales (*Delphinapterus leucas*) in Western Hudson Bay estuaries

**DOI:** 10.1371/journal.pone.0255756

**Published:** 2022-11-09

**Authors:** Kristin H. Westdal, Jeremy Davies, Steven H. Ferguson

**Affiliations:** 1 Faculty of Science, University of Manitoba, Winnipeg, Manitoba, Canada; 2 Oceans North, Ottawa, Ontario, Canada; 3 Ocean Conservancy, Bellingham, Washington, United States of America; 4 Fisheries and Oceans Canada, Freshwater Institute, Winnipeg, Manitoba, Canada; Texas A&M University, UNITED STATES

## Abstract

Segregation of adult males from adult females and immature animals is known to occur in some beluga whale populations, but it is unclear if such segregation occurs in Hudson Bay, where the largest summering population in the world is found. Using imagery from a photographic aerial survey conducted in August 2015, we examined spatial distribution by age class with respect to several environmental variables near two of three main estuaries, Churchill and Seal River, used by Western Hudson Bay belugas in the summer season. Belugas photographed during aerial surveys were classified by age manually using an identification decision tree, and GPS coordinates of their locations were plotted in ArcGIS. Distribution by age class was examined in relation to five habitat characteristics: distance to coastal habitat, bathymetry, sea surface temperature, and extent of river plume. Habitat characteristics and the proportion of animals by age classes were similar in both estuaries, indicating no segregation, and suggesting the environmental data assessed were not associated with patterns of distribution and density of age classes at the spatial and temporal scale being investigated. Overall density of calves was almost three times higher at the Seal River; however, suggesting this location may be preferred for calf rearing in the summer season. Results provide a greater understanding of spatial patterns of beluga whale habitat use in western Hudson Bay, and information useful in conservation and management advice.

## Introduction

Social and spatial segregation based on sex is common amongst whales that live in groups [1]; however, mysticetes and odontocetes are both known to segregate. Toothed whales are less solitary and more social between the two groups of whales [2] and likely show some sexual segregation. The North Atlantic right whale (*Eubalaena glacialis*), North Pacific gray whale (*Eschrichtius robustus*) and bowhead whale (*Balaena mysticetus*) are known to have segregated nursery areas where females feed and take care of their young [3–5]. Adult male sperm whales (*Physeter microcephalus*) separate from females, calves and juveniles outside of the mating season [6], and periodic segregation has been recorded in killer whales [7]. Narwhals, however, the closest relative of beluga, appear to travel in groups of mixed sex and age class [8].

**Fig 1:**
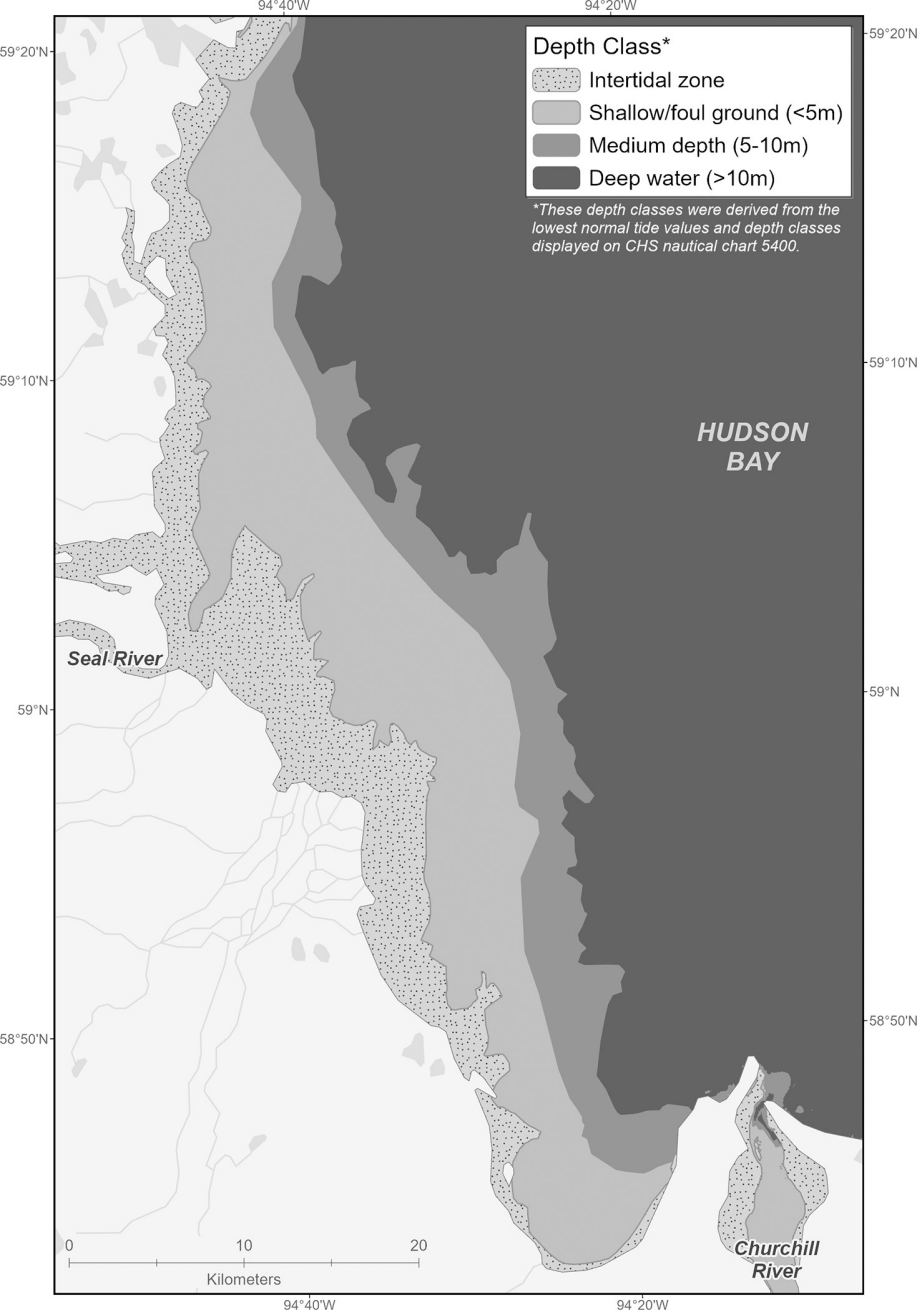
Seal River and Churchill River Estuary region with detailed depth classes. Data for depth classes republished from Natural Resources Canada CHS Chart 5400 under a CC BY license, with permission from Natural Resources Canada, original copyright 2003.

Segregation of adult male beluga whales (*Delphinapterus leucas*) from immature animals of both sexes is known to occur in belugas in the western Arctic [9], and habitat preferences differ for males and females in the central Arctic [10]; however, it is unclear if females accompanied by calves segregate for periods of time during the year in the eastern Arctic. Western Hudson Bay (WHB) beluga whales are smaller than individuals from other populations [11], and summer habitat is shallow [12] (Fig 1). In Hudson Bay, genetically related groups of beluga have been shown to migrate together, and females were found to be more likely to travel in a family group than adult males [13].

Western Hudson Bay belugas inhabit offshore areas with dense pack ice in winter, and prefer shallow warm water estuaries in the summer season (June-August). They undertake migrations of more than 2,000 km round trip between their summer and winter habitat, similar to long distance migrations of other beluga populations [14]. Although somewhat debated in the literature, hypotheses have been that estuaries are critical for calf rearing, and that they provide protection from predators, thermal advantage and an abundance of prey [9], but no study has examined the distribution and habitat use of females with calves in Western Hudson Bay.

In the western and central Canadian Arctic, male and female belugas have shown differing distributions, with females with calves assumed to be spending more time in shallow waters [10]. In the Beaufort Sea, belugas have been shown to segregate by length (age), sex, and reproductive status, where females with calves and small males select near-shore open water habitat, and larger males select ice covered offshore areas [15]. It is unknown, however, why these belugas segregate in the eastern Beaufort Sea but predation and foraging are two likely explanations [15]. In western Hudson Bay, foraging and predation may also explain beluga whale distribution.

Here our objectives were to determine if there is a difference in habitat association by female belugas with calves compared with other age classes, adults without calves and juveniles, in the Seal and Churchill River areas from aerial photos taken in 2015. In addition, we investigate differences in environmental characteristics that might explain differences in habitat use by age class.

## Methods

### Aerial survey

An analysis of the Fisheries and Oceans Canada (DFO) 2015 Western Hudson Bay beluga aerial survey was undertaken to look at belugas by age class, and their locations within the study area. The line transect survey took place between August 6 to 19, 2015, and followed methods and covered an area similar to that of Richard [16]. The survey covered an area beyond the known range of the Western Hudson Bay beluga, based on past surveys and satellite telemetry data [17]. A visual as well as photographic survey took place in high-density areas, allowing for individual identification of belugas. Here photos were analyzed from the Seal and Churchill river estuaries and surrounding area.

**Fig 2:**
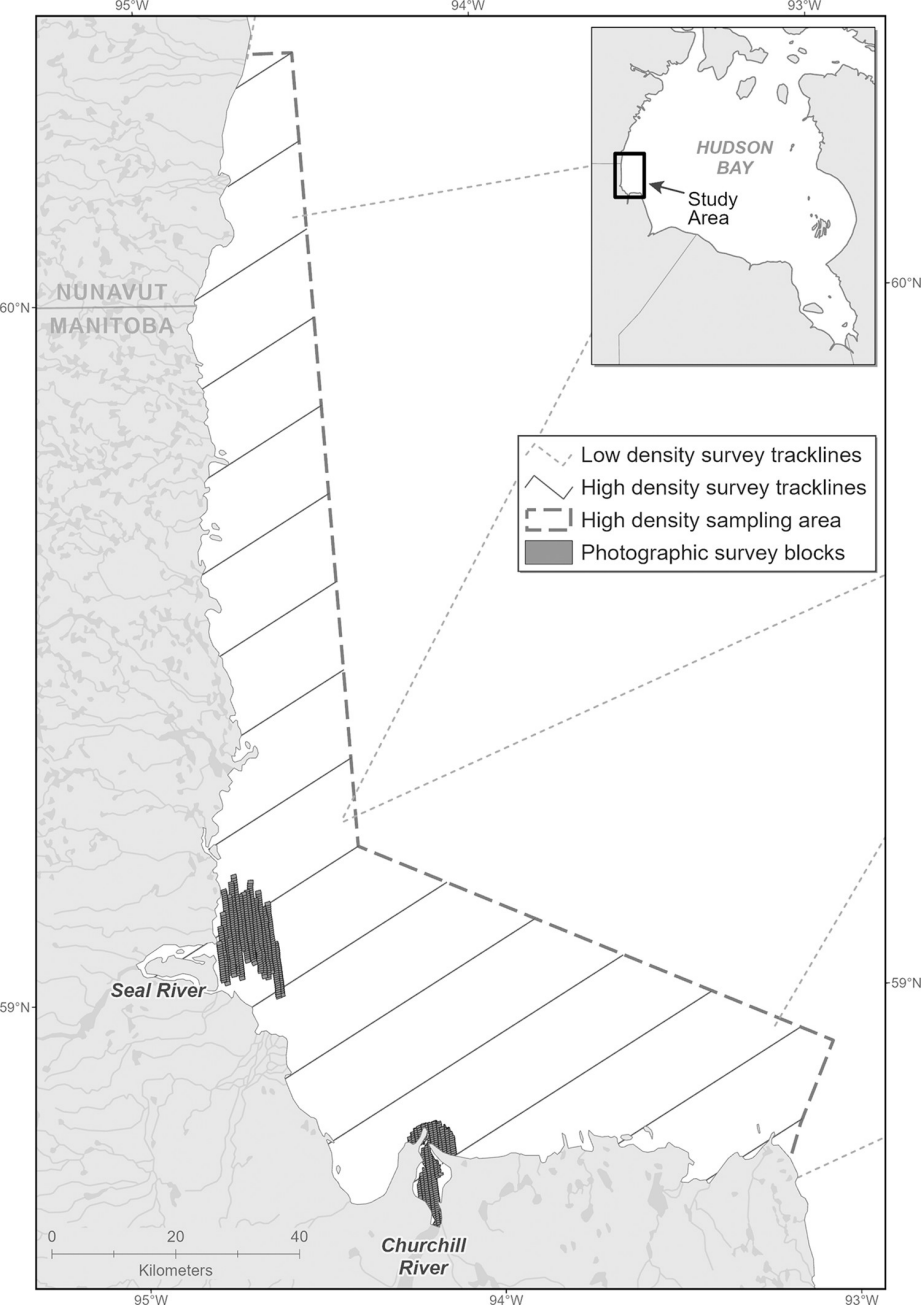
Western Hudson Bay 2015 beluga survey location detailing survey area, survey track lines and full photographic survey coverage.

Photographs were taken using two Nikon D810 cameras (25 mm and 50 mm lenses) mounted on a twin otter airplane flying at 2,000 feet over the Churchill and Seal River estuaries (complete photographic coverage on August 6, 12 and 19), high density areas (transect coverage on August 6,12, 18 and 19), and offshore low density areas (transect coverage on August 16) (Fig 2). For this analysis, complete photographic coverage from August 12 and 19 were used of both estuaries (amounting to one full survey of each) and transect coverage of high density areas was used from August 6 and 12. A review of photos from both cameras in the Churchill estuary determined that we were able to identify calves in photos from the 25 mm lens, and thus these photos were used in this analysis to provide more coverage than photos from the 50 mm lens. Individual photographs (n = 3,661) covered a surface area of approximately 857 x 585 m with an estimated 20–40% overlap between photos.

### Beluga classification

Photographs were scanned individually and belugas were identified and classified into three age categories (adult (without calf), juvenile, calf (accompanied by adult)) visually (Table 1). Calves and juveniles were distinguished from each other as well as adults using a variation on a dichotomous key developed to detect narwhal newborns in aerial photographs [18] combined with beluga characteristics [19, 20].

**Table 1:** Beluga whale age classification.

Age Class	Characteristics
**Adult**	Largest of three age classes
	White in colour
**Juvenile**	50–75% length of adult
	Gray in colour
**Calf**	33 to 50% length of adult
	< two body lengths away from adult whale
	Located adjacent to, on top or behind the nearest adult whale (i.e. not ahead of)

### Georeferencing and environmental variables

Photos were georeferenced, GPS coordinates of identified calves, juveniles and adults were plotted in ArcGIS, and duplicates (from overlapping photos) were removed. A grid with individual cells of 500m by 500m (0.25km^2^) in size was laid on top of the survey area and each individual was assigned a cell. Each grid cell containing belugas was designated as belonging to either Seal (n = 152), Churchill (n = 60), or "outside"(n = 11) (which was identified as beyond the full photographic survey of either estuary–see Fig 1), depending on the location. Total area for each of the three distinct locations was calculated by multiplying grid cell area by number of grid cells containing whales (i e. the total area where whales were found).

Sea surface temperature, distance to shore, distance to intertidal zone, distance to nearest river mouth and distance to river plume values were extracted to the center points (centroids) of each cell in the sampling grid. Spatial data were compiled and analyzed using ArcMap geoprocessing tools [21]. Distance to feature raster layers were generated for each of the input features (i.e., shoreline, intertidal zone, river mouth locations, and plume boundaries) using the ‘Euclidian Distance’ tool, and the values of each of variable were extracted to the centroids of each cell in the sampling grid using the ‘Extract Values to Points’ tool. The populated point layer was then exported as a table for further statistical analysis.

Average sea surface temperature data was obtained from the Group for High Resolution Sea Surface Temperature (GHRSST) [22], distance to shore, intertidal zone, and distance to nearest river mouth (center of river mouth from headland to headland) were calculated using Natural Resources Canada CHS Chart 5400 map as a base [23]. Plume extent data was sourced from the same month and year as the beluga survey data from MODIS High-resolution data from NASA [24]. Here turbidity data is used to delineate the river plume, and is measured by the diffuse attenuation coefficient of 490 nm (Kd490), which is a measure of light penetration in the water column. Kd490 is commonly used for assessing costal and turbid waters [25].

### Distribution testing

Proportion of belugas identified by age class was calculated for the three identified areas (Seal, Churchill and “Outside”), and Fisher’s Exact Test compared age class ratios between the two main estuaries, the Churchill and Seal River estuary. Median distance from the center of each cell to the closest boundary of each environmental feature and mean sea surface temperature within each cell were calculated for each estuary by age class. Beluga distribution by age class was examined in relation to distance to coastal habitat and bathymetry, sea surface temperature, and extent of river plume using Kruskal-Wallis test run in JASP [26]. Age class ratios were compared but distribution in relation to environmental features were not compared between estuaries due to the dissimilar coastlines and bathymetry.

## Results

**Fig 3:**
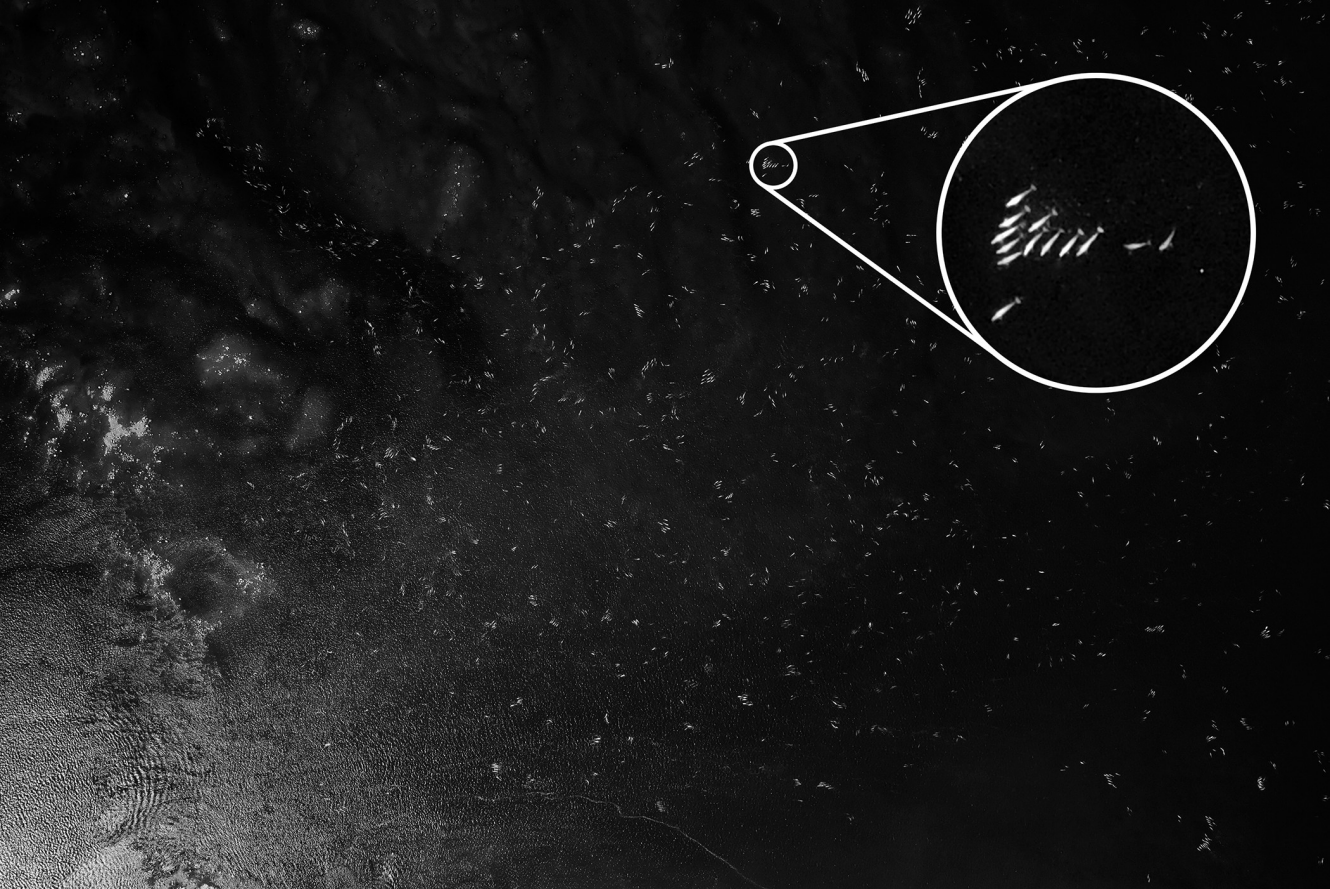
Western Hudson Bay 2015 survey photograph containing over 2,000 individual beluga whales.

The total number of belugas identified, after removing duplicates from overlapping photos, was 13,538 (Table 2). Dense clumping of animals was common at the two estuaries, particularly near the Seal River Estuary (Fig 3), where over 2,000 individuals in 0.5km^2^ could be observed in one location.

**Table 2:** Total beluga whales identified, proportion of total and density per km^2^ in 2015 Western Hudson Bay survey (not including the Nelson River Estuary) by location and age class.

	Location			
Age Class	Seal	Churchill	“Outside”	Total number of animals
Adult (without calf)				
*Count*	11,115	1,031	106	12,252
*Percent of total*	(90.6%)	(89.4%)	(88.3%)	
*Area*	38km^2^	15km^2^	2.75km^2^	
*Density per km* ^*2*^	293	69	39	
Juvenile				
*Count*	629	52	6	687
*Percent of total*	(5.1%)	(4.5%)	(5%)	
*Area*	38km^2^	15km^2^	2.75km^2^	
*Density per km* ^*2*^	17	3	2	
Calf				
*Count*	521	70	8	599
*Percent of total*	(4.3%)	(6.1%)	(6.7%)	
*Area*	38km^2^	15km^2^	2.75km^2^	
*Density per km* ^*2*^	14	5	3	
Total	12,265	1,153	120	13,538

Beluga numbers were highest at the Seal Estuary (n = 12,265), with few animals identified offshore (n = 120) (Table 2). Overall area where belugas were identified was highest at the Seal Estuary as well as density, number of belugas per square kilometer. The Churchill and the Seal estuaries contained a similar proportion of beluga by age class. Calves made up 4.25% to 6.67% of the total beluga count in both estuaries, as well as the small number of belugas clumped between the two estuaries (Table 2).

Fisher’s Exact Test was used to determine if there was a significant difference in age class ratios between the Seal and Churchill estuaries. There was not a statistically significant difference between the Seal and Churchill estuaries (p = 0.936), confirming similarity of age classes between estuaries (Table 2).

**Fig 4:**
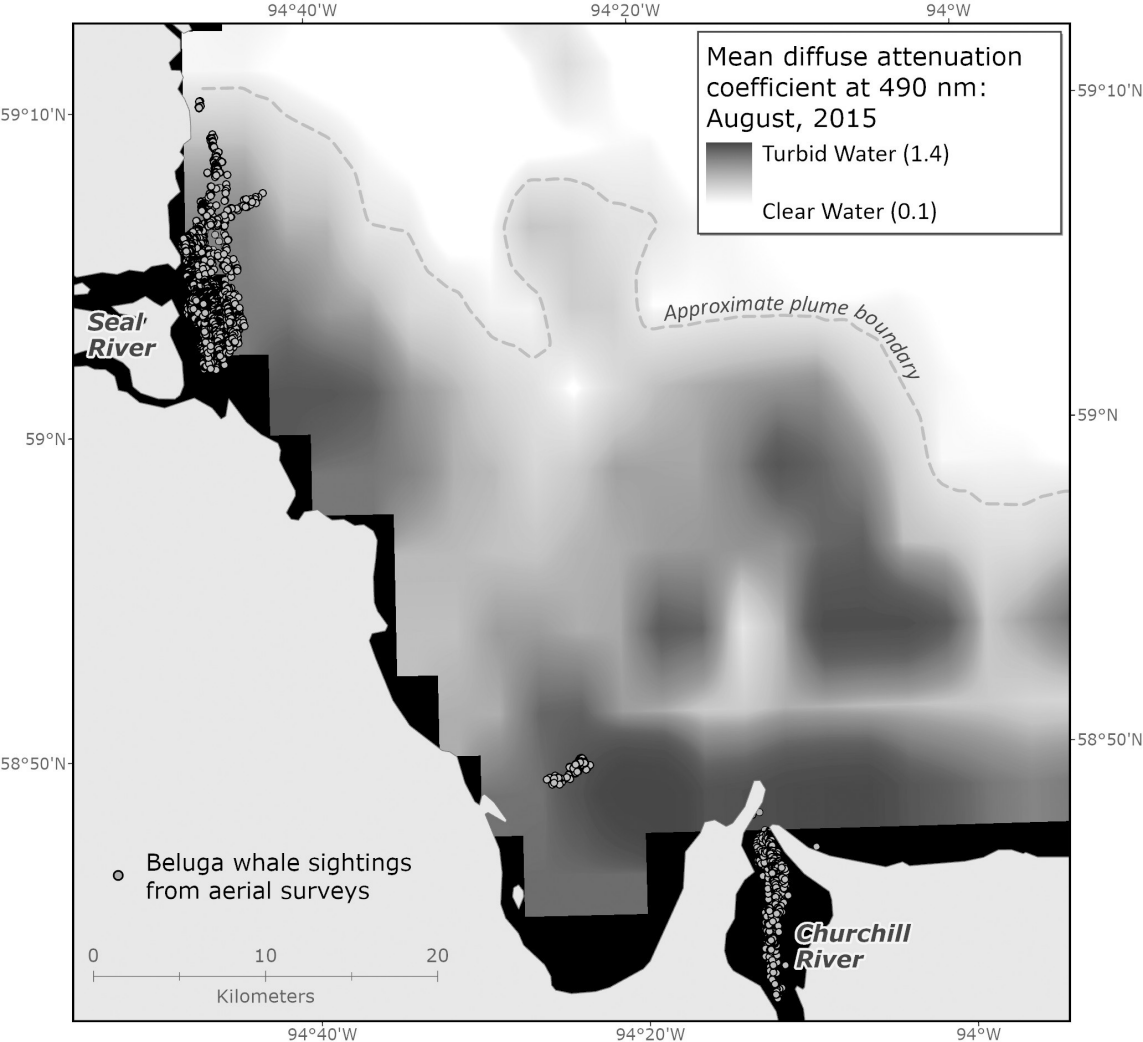
Beluga locations identified from 2015 aerial survey in southwest Hudson Bay overlaid on the estimated plume extent for the month of August, 2015 (NASA, 2018). [Approximate plume boundary defined using the 0.3 m-1 isopleth, which is the KD490 value that corresponds with the gradient between turbid and clear water, as shown on the map. The black blocks represent no-data areas in KD490 dataset.].

Median distance to the intertidal zone, coast and river were calculated for each estuary by age class as well as median sea surface temperature (Table 3). Distances to geographical features did not differ by age class for either estuary (Table 3). Median sea surface temperatures were similar for all age classes at both locations (Table 3). All belugas observed were inside the river plume extent (Fig 4).

**Table 3:** Belugas by estuary and age class compared to environmental variables: Median distance (25% and 75% quantile) to intertidal zone, distance to coast, distance to river plume, distance to river and sea surface temperature.

Age Class	Median distance (m) to intertidal zone		Median distance (m) to coast		Median distance (m) to river plume		Median distance (m) to river		Median SST (°C)	
	Churchill	Seal	Churchill	Seal	Churchill	Seal	Churchill	Seal	Churchill	Seal
*Adult*	**600**	**0**	**825**	**1,204**	**14,342**	**12,298**	**3,114**	**2,631**	**12.38**	**12.14**
	Q1: 316	Q1: 0	Q1: 447	Q1: 640	Q1: 13,354	Q1: 11,241	Q1: 2,110	Q1: 2,102	Q1: 12.34	Q1: 12.12
	Q3: 806	Q3: 0	Q3: 1,118	Q3: 1,703	Q3: 16,224	Q3: 12,971	Q3: 4,105	Q3: 3,289	Q3: 12.44	Q3: 12.15
	n = 1031	n = 11,115	n = 1031	n = 11,115	n = 1031	n = 11,115	n = 1031	n = 11,115	n = 1031	n = 11,115
*Juvenile*	**550**	**0**	**825**	**1,400**	**14,812**	**12,093**	**2,861.50**	**2,631**	**12.38**	**12.14**
	Q1: 408.25	Q1: 0	Q1: 435.25	Q1:707	Q1: 13,354	Q1:11,374	Q1: 1,989.50	Q1:2,102	Q1: 12.34	Q1:12.12
	Q3: 825	Q3: 0	Q3: 1,087.25	Q3:1,709	Q3:15,856.25	Q3:12,777	Q3: 4,105	Q3:3,228	Q3: 12.43	Q3:12.15
	n = 52	n = 629	n = 52	n = 629	n = 52	n = 629	n = 52	n = 629	n = 52	n = 629
*Calf*	**500**	**0**	**825**	**1,476**	**14,821**	**12,093**	**2,617**	**2631**	**12.38**	**12.13**
	Q1: 316	Q1: 0	Q1: 447	Q1: 900	Q1: 13,354	Q1:11,180	Q1: 1,668.25	Q1: 2138	Q1: 12.34	Q1:12.12
	Q3: 806	Q3: 0	Q3: 1107.75	Q3: 1900	Q3:16,092.75	Q3:12,777	Q3: 4,371.75	Q3: 3289	Q3: 12.45	Q3:12.15
	n = 70	n = 521	n = 70	n = 521	n = 70	n = 521	n = 70	n = 521	n = 70	n = 521

Kruskal-Wallis test comparing differences between environmental variables and age class indicated a significant difference between age classes and distance to coast near the Seal River (p = <0.001) (Table 4).

**Table 4:** Kruskal-Wallis test comparing differences between environmental co-variates and age class (adult, juvenile, calf) of belugas near the Churchill and Seal River Estuaries.

		Statistic	df	p
**Intertidal**	Churchill	0.367	2	0.83
	Seal	0.867	2	0.65
**Distance to Coast**	Churchill	0.136	2	0.93
	Seal	23.311	2	<0.001*
**Distance to River**	Churchill	0.548	2	0.76
	Seal	2.101	2	0.35
**Sea Surface Temperature**	Churchill	0.333	2	0.85
	Seal	1.423	2	0.49

## Discussion

The Western Hudson Bay beluga population gathers in dense groups in the summer season. We found that within these dense groups, females with calves did not select separate habitat from juveniles and or adults without calves. Our analysis considered five environmental factors that helped assess commonly held hypotheses as possible drivers of distribution, and found that none of these factors was significantly associated with beluga age class. All beluga groups were observed well inside the river plume extent, sea surface temperatures were similar across the study area, and distances to geographical features were similar across age classes by estuary. Similar to our findings, Kelley and Ferguson [27] found that male and female belugas (from Eastern and Western Hudson Bay beluga populations), in and out of mating season, appeared to be foraging similarly and perhaps occupying the same habitat; however Barber et al. [10] (Eastern Beaufort Sea and Eastern High Arctic-Baffin Bay) and Loseto et al. [15] (Eastern Beaufort Sea) both found segregation of belugas by age or sex. This is perhaps an indication that Hudson Bay belugas are different in factors other than being of a smaller body size from other Canadian beluga populations.

Proportion of belugas by age classes were similar between the Churchill and Seal River estuaries, and the cluster of animals between the estuaries. A range of 4.3%-6.1% of visible whales were calves across the three identified regions; however, overall density of calves was almost three times higher at the Seal River suggesting this location may be preferred for calf rearing in the summer season. Exhibiting high site fidelity, belugas are thought to return to the same locations seasonally each year [28]. In this region, individual belugas identified by markings have been resighted year to year (S. Peterson, personal communication, K. Westdal, personal observations), and recent Western Hudson Bay beluga population estimates (2004 and 2015) are also similar (57,300 (95% CI: 37,700–87,100) [30], 54,473 (95% CI: 44,988–65,957) [17].

Distance to key environmental features did not differ among age classes, save for distance to coast at the Seal River, which overall suggests a mixed pattern of distribution amongst age classes. All belugas were observed inside the river plume, sea surface temperature was similar across the study area, and distances to land and or bathymetric locations were not significant across age classes. A detailed look at distance to coast for belugas identified near the Seal River estuary shows that the mean distance for adults is not that dissimilar to calves and juveniles; however the distribution was less uniform. Bathymetry in this region could explain the variation with mud and tidal flats extending greater than 10 km offshore non-uniformly. All age classes were observed in the same habitat type, intertidal and shallow or foul ground (less than 3 meters at average low tide) despite the variation. This bathymetry, which is different from that near the Churchill Estuary, did not allow for a comparison from one estuary to another.

This study offers little evidence to support the main hypotheses on estuary use. We did not see selection of habitat based on bathymetry and proximity to shore for females with calves more frequently than other age classes, which would lend itself to the predation risk hypothesis. The predation risk hypothesis suggests that female belugas with calves would prefer shallower waters [29] where killer whales would be excluded (although belugas would still have access to this habitat), lowering risk of mortality for their offspring in the summer season [30, 31]. We did not see any evidence pointing to thermal advantage given that sea surface temperature was nearly uniform across the study area. This reinforces that selection of warmer waters is not necessary for beluga neonates as suggested by Doidge et al. [32]. Other cetaceans, such as gray whales, may select warmer waters for their calves, who have limited blubber at birth [33]. Beluga calves, however, are thought to have thick skin with a blubber layer at birth to protect them from colder waters [34], which questions the thermal advantage hypothesis. Thermal advantage, however, has been supported as a driver of beluga habitat use in the Western Canadian Arctic [35]. In Western Hudson Bay, we would expect to find females with calves in warmer waters than other age groups if thermal advantage was a driver of distribution. Selection of shallow waters may play a role in habitat selection, but unlikely to be the significant determinant (as suggested by Smith et al. [9]) based on the distribution of belugas and lack of differentiation seen amongst age classes. All animals were also well inside the river plume that presumably offered more forage fish as prey [36], which does not suggest the forage-selection hypothesis is central to habitat selection. The forage-selection hypothesis assumes that the belugas are choosing this particular habitat based on energetic requirements. Larger animals (males, in the case of belugas) and females nursing young require more or higher quality prey [37]. We might not expect to see males and females with young calves in different locations but selecting similar habitats that provides good foraging opportunities, such as estuarine waters and the river plume [36].

The highly social nature of belugas [38], like other cetaceans, may make it difficult to understand abundance and distribution in relation to environmental characteristics [39]. Marine mammals are known to congregative in large aggregations that are not clearly tied to habitat [39]. Beluga may be gathering in large groups in the summer season for social rather than environmental reasons. Rendell and Whitehead [40] suggest that particular locations may be selected seasonally as a result of cultural traditions. Beluga migration routes and seasonal habitat association appear to be passed down through females to offspring in belugas [41].

Uncertainties remain, however, based on environmental data available and nature of the beluga photographic survey data. We were also using data from one survey in one year, providing a snapshot in time, in an estuarian region that is ephemeral in nature. Anderson et al. [42] for example found that beluga groups with calves in Cunningham Inlet were found closer to shore on cloudier days (possible terrestrial anti-predation behaviour). And Smith et al. [9] found that beluga distribution from the Nelson River mouth varied depending on fresh water outflow volumes from the river by year. Prey distribution and salinity were not included as environmental variables due to a lack of data availability. The nature of the clumping of belugas also made analysis options limited. The key variable to distribution may be social behaviour no matter what environmental data we have to work with.

Future studies could include collection of prey and salinity data, focal follow studies focusing on females and calves, as well as longer time-frame video monitoring to look at group structure over time. Further understanding is needed of social behaviour and habitat use over the full summer season, as well as age class distribution in the third major estuary utilized by beluga in the region (i.e., Nelson River estuary). Our results provide new information on beluga habitat use for conservation and management planning, particularly on marine protection boundary delineation, in the light of provincial government interest in protection of upstream habitat and Federal government interest in coastal marine protection in Western Hudson Bay.

## Supporting information

S1 File CHS copyright confirmation. (DOCX)

S2 File (PDF)

## References

[1] Ruckstuhl KE, Neuhaus P. Sexual segregation in vertebrates: ecology of the two sexes. Cambridge University Press. 2005. doi: 10.1017/CBO9780511525629

[2] Wade PR, Reeves RR, Mesnick SL. Social and behavioural factors in cetacean responses to overexploitation: Are odontocetes less “resilient” than mysticetes? Journal of Marine Sciences, vol. 2012, Article ID 567276, 15 pages, 2012. 10.1155/2012/567276.

[3] Cosens SE, Blouw A. Size and age class segregation of bowhead whales summering in northern Foxe Basin: A photogrammetric analysis. Mar Mamm Sci. 2006;19(2): 284–296.

[4] Hamilton PK, Cooper LA. Changes in North Atlantic right whale (Eubalaena glacialis) cow-calf association times and use of the calving ground: 1993–2005. Mar. Mamm. Sci. 2010;26(4): 896–916.

[5] Urban RJ, Rojas-Bracho L, Perez-Cortes H, Gomez-Gallardo A, Swartz SL, Ludwig S, et al. A review of gray whales (Eschrichtius robustus) on their wintering grounds in Mexican waters. J. Cetacean Res. Manage. 2003;5(3):281–295.

[6] Christal J, Whitehead H. Social affiliations within sperm whale (*Physeter microcephalus*) group. Ethology. 2001;107:323–240.

[7] Beerman A, Ashe E, Preedy K, Williams R. Sexual segregation when foraging in an extremely social killer whale population. Behavioral ecology and sociobiology. 2016; 70(1):189–198.

[8] Marcoux M, Auger-Methe M, Humphries MM. Encounter frequencies and grouping patterns of narwhals in Koluktoo Bay, Baffin Island. Polar Biology. 2009;32:1705–1716.

[9] Smith AJ, Higdon JW, Richard P, Orr J, Bernhardt W, Ferguson SH. Beluga whale summer habitat associations in the Nelson River estuary, Western Hudson Bay, Canada. PLoS ONE. 2017;12(8): doi: 10.1371/journal.pone.0181045 28767655 PMC5540293

[10] Barber DG, Saczuk E, Richard PR. Examination of beluga-habitat relationships through the use of telemetry and a Geographic Information System. Arctic. 2001;54:305–316.

[11] Ferguson SH, Yurkowski D, Hudson J, et al. Larger body size leads to greater female beluga fitness at the southern periphery of their range. Authorea. December 01, 2020. doi: 10.22541/au.160682236.63502812/v1 PMC8668808 34938510

[12] Martin AR, Hall P, Richard PR. Dive behaviour of belugas (*Delphinapterus leucas*) in the shallow waters of Western Hudson Bay. 2001;54(3):276–283.

[13] Colbeck GJ, Duchesne P, Postma LD, Lesage V, Hammill MO, Turgeon J. Groups of related belugas (Delphinapterus leucas) travel together during their seasonal migrations in and around Hudson Bay. Proc R Soc. 2013;280. doi: 10.1098/rspb.2012.2552 23222451 PMC3574313

[14] Luque SP, Ferguson SH. Age structure, growth, mortality, and density of belugas (Delphinapterus leucas) in the Canadian Arctic: responses to environment? Polar Biology. 2010;33(2):163–178.

[15] Loseto LL, Richard P, Stern GA, Orr J, Ferguson SH. Segregation of Beaufort Sea beluga whales during the open water season. Can. J. Zool. 2006;84:1743–1751.

[16] Richard PR. An estimate of the western Hudson Bay beluga population size in 2004. Canadian Science Advisory Secretariat Research Document 2005/017.

[17] Matthews CJD, Watt CA, Asselin NC, Dunn JB, Young BG, Montsion LM, et al. Estimated abundance of the Western Hudson Bay beluga stock from the 2015 visual and photographic aerial survey. DFO Can. Sci. Advis. Sec. Res. Doc. 2017/061. v + 20 p.

[18] Charry B, Marcoux M, Humphries MH. Aerial photographic identification of narwhal (Monodon monoceros) newborns and their spatial proximity to the nearest adult female. Arctic Science. 2018;4:513–524

[19] Brodie PF. A reconsideration of aspects of growth, reproduction, and behavior of the white whale (*Delphinapterus leucas*), with reference to the Cumberland Sound, Baffin Island, population. Journal of the Fisheries Board of Canada. 1971;28:1309–1318.

[20] Michaud R. St. Lawrence Estuary beluga (*Delphinapterus leucas*) population parameters based on photo-identification surveys, 1989–2012. DFO Canadian Science Advisory Secretariat Research Document. 2104;2013/130.

[21] Esri Inc. ArcMap (version 10.7.1). Software. Redlands, CA: Esri Inc, 2019.

[22] JPL OurOcean Project. 2010. GHRSST Level 4 G1SST Global Foundation Sea Surface Temperature Analysis. Ver. 1. PO.DAAC, CA, USA. Dataset accessed [2020-03-10] at 10.5067/GHG1S-4FP01

[23] Canadian Hydrographic Service. Chart 5400. Cape Churchill to/à Egg River. 2003 edition. Fisheries and Oceans Canada, Government of Canada. Ottawa, Ontario. User Licence no. 2021-0723-1260-O.

[24] NASA Goddard Space Flight Center, Ocean Ecology Laboratory, Ocean Biology Processing Group; (2018): Diffuse attenuation coefficient at 490 nm, Ocean Color Data, NASA OB.DAAC. https://oceancolor.gsfc.nasa.gov/l3/. Accessed on 2021/11/18.

[25] Shi W, Wang M. Characterization of global ocean turbidity from Moderate Resolution Imaging Spectroradiometer ocean color observations. Journal of Geophysical Research. 2010;115(C11):1–14.

[26] JASP Team. JASP (Version 0.14.1) 2020 [Computer software]

[27] Kelley TC, Ferguson SH. Sexual segregation in two closely related species: Beluga whales (Delphinapterus leucas) and narwhal (Monodon monoceros). PhD Thesis, The University of Manitoba. 2014. Available from: https://mspace.lib.umanitoba.ca/bitstream/handle/1993/23548/kelley_tritsya.pdf?sequence=1

[28] COSEWIC. COSEWIC assessment and status report on the Beluga Whale Delphinapterus leucas, Eastern High Arctic—Baffin Bay population, Cumberland Sound population, Ungava Bay population, Western Hudson Bay population, Eastern Hudson Bay population and James Bay population in Canada. Committee on the Status of Endangered Wildlife in Canada. 2020 In Press. Ottawa. xxxv + 84 pp.

[29] Grignolio S, Rossi I, Bassano B, Apollonio M. Predation risk as a factor affecting sexual segregation in Alpine Ibex. Journal of Mammalogy. 2007;88(6):1488–1497. doi: 10.1644/06-MAMM-A-351R.1

[30] Main M.B., Weckerly F.W., and Bleich V.C. Sexual ssegregation in ungulates: new directions for research. Journal of Mammology. 1996;77:449–461.

[31] Westdal KH, Davies J, MacPherson A, Orr J, Ferguson SH. Behavioural changes in Belugas (Delphinapterus leucas) during a Killer Whale (Orcinus orca) attack in southwest Hudson Bay. Canadian Field-Naturalist. 2016;130(4):315–319.

[32] Doidge DW. Integumentary heat loss and blubber distribution in the beluga, Delphinapterus leucas, with comparisons to the narwhal, *Monodon monoceros*. In: Smith TG, St. Aubin DG, Geraci JR, editors. Advances in research on the beluga whale, Delphinapterus leucas. Can Bull Fish Aquat Sci; 1990. pp.129–140.

[33] Clapham PJ. Why do baleen whales migrate? A response to Corkeron and Connor. Marine Mammal Science. 2001;17:432–436.

[34] Doidge D.W. 1990a. Age-length and length-weight comparisons in the beluga, Delphinapterus leucas. In: Smith T.G., St.Aubin D.J.,. and Geraci J.R. Eds. Advances in research on the beluga whale, Delphinapterus leucas. Canadian Bulletin of Fisheries and Aquatic Sciences 224. p. 59–68.

[35] Scharffenberg K, Whalen D, Marcoux M, Iacozza J, Davoren G, Losteo L. Environmental drivers of beluga whale (*Delphinapterus leucas*) habitat use in the MacKenzie Estuary, Northwest Territories, Canada. Marine Ecology Progress Series. 2019;626:209–226.

[36] Huntington HP and the communities of Buckland, Elim, Koyuk, Point Lay, and Shaktoolik. Traditional Knowledge of the Ecology of Beluga Whales (*Delphinapterus leucas*) in the Eastern Chukchi and Northern Bering Seas, Alaska. Arctic. 1999;52(1):49–61.

[37] Braithwaite JE, Meeuwig JJ, Hipsey MR. Optimal migration energetics of humpback whales and the implications of disturbance. Conservation Physiology. 2015;3(1):1–15. doi: 10.1093/conphys/cov001 27293686 PMC4778463

[38] Kingsley M, Gosselin S, Sleno G. Movements and Dive Behaviour of Belugas in Northern Quebec. Arctic. 2001;54(3):262–275.

[39] Whitehead H, Parijs SV. Studying marine mammal social systems. In: Boyd I, Bowen D, Iverson S, editors. Marine mammal ecology and conservation: a handbook of techniques. Oxford University Press, Oxford, U.K.; 2010. pp.263–282.

[40] Rendell L, Whitehead H. Culture in whales and dolphins, Behav. Brain Sci, 2001;24:309–382. doi: 10.1017/s0140525x0100396x 11530544

[41] O’Corry-Crowe GM, Suydam RS, Rosenberg A, Frost KJ, Dizon AE. Phylogeography, population structure and dispersal patterns of the beluga whales (*Delphinapterus leucas*) in the western Nearctic revealed by mitochondrial DNA. Molecular Ecology. 1997;6: 955–970.

[42] Anderson PA, Poe RB, Thompson LA, Webber N, Romano TA. 2017. Behavioral responses of beluga whales (*Delphinapterus leucas*) to environmental variation in an Arctic estuary. Behav. Process. 2017;145:48–59. 10.1016/j.beproc.2017.09.007 28927964

