# Supplementary material for: Assessing seasonal spatial segregation by age class of beluga whales (Delphinapterus leucas) in Western Hudson Bay estuaries
Source: PLoS One. 2022 Nov 9;17(11):e0255756. doi: 10.1371/journal.pone.0255756 (PMC9645605; doi:10.1371/journal.pone.0255756)
Supplement: S1 File — (DOCX) [file pone.0255756.s001.docx]

**From:** Holroyd, Paul <[Paul.Holroyd@dfo-mpo.gc.ca](mailto:Paul.Holroyd@dfo-mpo.gc.ca)>
**Sent:** Monday, March 7, 2022 6:01:39 AM
**To:** Jeremy Davies <[jdavies@oceanconservancy.org](mailto:jdavies@oceanconservancy.org)>
**Cc:** Blanchard, Jennifer <[Jennifer.Blanchard@dfo-mpo.gc.ca](mailto:Jennifer.Blanchard@dfo-mpo.gc.ca)>
**Subject:** RE: Request for CHS Intellectual Property Licence (#2021-0723-1260-O)

**CAUTION:** This e-mail originated from outside of Ocean Conservancy. Do not click on links or open attachments unless you recognize the sender and know that the content is safe.

Hi Jeremy – Yes, that’s fine if you’re one of the authors and if you’re the only one receiving and using the CHS data.

Cheers,

Paul

**From:** Jeremy Davies <[jdavies@oceanconservancy.org](mailto:jdavies@oceanconservancy.org)> 
**Sent:** March 4, 2022 2:07 PM
**To:** Holroyd, Paul <[Paul.Holroyd@dfo-mpo.gc.ca](mailto:Paul.Holroyd@dfo-mpo.gc.ca)>
**Cc:** Blanchard, Jennifer <[Jennifer.Blanchard@dfo-mpo.gc.ca](mailto:Jennifer.Blanchard@dfo-mpo.gc.ca)>
**Subject:** RE: Request for CHS Intellectual Property Licence (#2021-0723-1260-O)

Hi again—I assumed that it is ok that just I signed a license agreement with CHS, but it just occurred to me that you may require any co-authors of published science papers that include data derived from Chart 5400 to sign as well? Hopefully it is fine having just me as a licensed user, since I’m the one who does all the spatial analyses and mapping.

Thanks-- Jeremy

**From:** Jeremy Davies 
**Sent:** Friday, March 4, 2022 9:13 AM
**To:** Holroyd, Paul <[Paul.Holroyd@dfo-mpo.gc.ca](mailto:Paul.Holroyd@dfo-mpo.gc.ca)>
**Cc:** Blanchard, Jennifer <[Jennifer.Blanchard@dfo-mpo.gc.ca](mailto:Jennifer.Blanchard@dfo-mpo.gc.ca)>
**Subject:** RE: Request for CHS Intellectual Property Licence (#2021-0723-1260-O)

Hi Paul—thanks for the clarification and for this simple solution! This puts this issue to rest and my mind at ease.

I officially and formally affirm that we mutually agree to amending the purpose of agreement 2021-0723-1260-O to include publishing products derived from the CHS Charts and/or data in scientific journals.

Best wishes,

---

Jeremy Davies

Marine Conservation Geographer

Ocean Conservancy – Arctic Program

360.820.1212

**From:** Holroyd, Paul <[Paul.Holroyd@dfo-mpo.gc.ca](mailto:Paul.Holroyd@dfo-mpo.gc.ca)> 
**Sent:** Friday, March 4, 2022 7:44 AM
**To:** Jeremy Davies <[jdavies@oceanconservancy.org](mailto:jdavies@oceanconservancy.org)>
**Cc:** Blanchard, Jennifer <[Jennifer.Blanchard@dfo-mpo.gc.ca](mailto:Jennifer.Blanchard@dfo-mpo.gc.ca)>
**Subject:** RE: Request for CHS Intellectual Property Licence (#2021-0723-1260-O)

**CAUTION:** This e-mail originated from outside of Ocean Conservancy. Do not click on links or open attachments unless you recognize the sender and know that the content is safe.

Hi Jeremy – That’s a very good question and I appreciate you raising it.  I can clarify and offer a solution to help you.

Clauses 3, 4 and 5(c) limit you to the purpose stated in your application, which is to “use the chart in studies of various species distributions as related to depth and bottom type in the vicinity of the Churchill and Seal rivers in Hudson Bay.”  There is no publication permission there.  That said, clause 5(b) provides that you acknowledge CHS in any publication derived from the CHS charts and shows the note that is required (or any other notices approved in writing in advance by CHS).  So the question is how do you go about getting permission to add the creation of derived products to the purpose.

The solution to this to address the concerns raised by your journal editor on the use of your product derived from the chart can be addressed using clause 9, the amending clause, of the DULA.  This can simply be covered by this exchange of email, i.e. by you responding affirmatively that we mutually agree to amending the purpose of agreement 2021-0723-1260-O to include publishing products derived from the CHS Charts and/or data in scientific journals.

I hope this addresses the concerns of you and you journal editor and I look forward to your reply.

Kind regards,

**Paul N. Holroyd**

Special Advisor, Intellectual Property and Licensing, Canadian Hydrographic Service    Conseiller Spécial, Proprieté Intellectuelle et Licences, Service hydrographique du Canada

Fisheries & Oceans Canada, Government of Canada                                                      Pêches et Océans Canada, Gouvernement du Canada

**From:** Jeremy Davies <[jdavies@oceanconservancy.org](mailto:jdavies@oceanconservancy.org)> 
**Sent:** March 3, 2022 2:26 PM
**To:** Blanchard, Jennifer <[Jennifer.Blanchard@dfo-mpo.gc.ca](mailto:Jennifer.Blanchard@dfo-mpo.gc.ca)>; Holroyd, Paul <[Paul.Holroyd@dfo-mpo.gc.ca](mailto:Paul.Holroyd@dfo-mpo.gc.ca)>
**Subject:** RE: Request for CHS Intellectual Property Licence (#2021-0723-1260-O)

Hello Jenifer and Paul—I purchased the pdf version of Chart 5400 from CHS last July, and have attached the signed license agreement. I am a co-author on a paper we submitted to PLOS ONE that includes a map which displays depth bin data that are based on the depths displayed on the chart (see attached figure), and the journal editor believes that this a copyright or licensing violation. My understanding is that since the data I generated and mapped for this figure are BASED on a licensed copy of CHS Chart 5400 but I don’t display the actual chart, I am not violating and ownership or licensing agreements. Can you please tell me if I am correct in my understanding? I know the DULA does not allow sharing or publishing of the actual chart. If displaying data derived from a CHS chart as I have done in the attached figure is also off-limits, I will honor that and need to find some other way to map this information.

Thanks for any insight you can provide.

Sincerely,

---

Jeremy Davies

Ocean Conservancy – Arctic Program

360.820.1212
