# Supplementary material for: Assessing seasonal spatial segregation by age class of beluga whales (Delphinapterus leucas) in Western Hudson Bay estuaries
Source: PLoS One. 2022 Nov 9;17(11):e0255756. doi: 10.1371/journal.pone.0255756 (PMC9645605; doi:10.1371/journal.pone.0255756)
Supplement: S2 File — (PDF) [file pone.0255756.s002.pdf]

Request for Permission to Publish Content under CC-BY License

Dear Rights Holder or Representative,

I have submitted a paper for publication in a PLOS journal, and wish to include the content listed below in the paper. I'm hereby requesting your (or your company's or institution's) permission to include the content in my paper. Please note that all PLOS journals are published under a Creative Commons Attribution License (CC BY), which allows for unrestricted use and distribution, even commercial, as long as attribution is given to the creator or rights holder of the content. See https://creativecommons.org/licenses/by/4.0/.

To grant me permission to use the content in my PLOS paper, please fill in the information below and then scan the completed form and send it to me at my email address.

Thank you.

My name:

Jeremy Davies

My email address:
jdavies@oceanconservancy.org

Description of the content which I'm seeking permission to use (citation and/or title, and pasted screen shot, if applicable):

Figure 1 of submission PONE-D-21-23772R1, which is a map that shows depth bins that are based on data displayed on CHS Chart 5400.

Link to the Content:

https://charts.gc.ca/charts-cartes/nautical-marines-eng.html

* * *

On behalf of myself or the rights holder, I hereby grant the permission sought herein.

Signature of Party Granting Permission:

Geneviève Béchard Signature numérique de Geneviève Béchard
Date : 2022.03.28 14:14:21 -04'00'

Date:

Printed Name and Title:

Director General, Canadian Hydrographic Service and Hydrographer General of Canada
